# circNFATC3 facilitated the progression of oral squamous cell carcinoma via the miR-520h/LDHA axis

**DOI:** 10.1515/med-2023-0630

**Published:** 2023-06-27

**Authors:** Hongguo Xie, Xiaopeng Lu

**Affiliations:** Department of Stomatology, Jingmen No. 1 People’s Hospital, No. 168, Xiangshan Avenue, Duodao District,, Jingmen, 448000, Hubei, China; Department of Stomatology, Jingmen No. 1 People’s Hospital, Jingmen, 448000, Hubei, China

**Keywords:** oral squamous cell carcinoma, circNFATC3, miR-520h, LDHA, glycolysis

## Abstract

The aim of this study was to verify the effects of circular RNA nuclear factor of activated T-cells, cytoplasmic 3 (circNFATC3), in oral squamous cell carcinoma (OSCC) development. The levels of circNFATC3, microRNA-520h (miR-520h), and lactate dehydrogenase A (LDHA) were measured by qRT-PCR and western blot analysis. The cellular functions were assessed by using commercial kits, MTT assay, EdU assay, flow cytometry analysis, and transwell assay. The interactions between miR-520h and circNFATC3 or LDHA were confirmed by dual-luciferase reporter assay. Finally, the mice test was enforced to evaluate the character of circNFATC3. We observed that the contents of circNFATC3 and LDHA were upregulated and miR-520h levels were downregulated in OSCC tissues compared with those in paracancerous tissues. For functional analysis, circNFATC3 knockdown repressed the cell glycolysis metabolism, cell proliferation, migration, and invasion, although it improved cell apoptosis in OSCC cells. LDHA could regulate the development of OSCC. circNFATC3 acted as a miR-520h sponge to modulate LDHA expression. In addition, the absence of circNFATC3 subdued tumor growth *in vivo*. In conclusion, circNFATC3 promoted the advancement of OSCC by adjusting the miR-520h/LDHA axis.

## 1 Introduction

Oral squamous cell carcinoma (OSCC) is a severe problem globally because of its most serious influence on the life quality of patients [1,2,3]. The survival rate of OSCC patients is merely 40–50% [4]. Although clinical treatment methods like surgical methods and chemoradiotherapy have been improved in decades, the treatment consequence is still unsatisfactory. Consequently, it is urgently indispensable to discover new therapies, especially targeting accurate pathogenesis. In recent years, molecular targeted therapy has developed gradually, but it is not mature. The specific mechanism also needs further study.

Circular RNAs (circRNAs) are a kind of regulatory RNAs, which are categorized by forming closed-loop structures through a covalent bond and could be stably present in organisms [5]. It has been reported that numerous circRNAs are specifically expressed in various tissues in humans and diversely expressed in cancer versus normal tissues, insinuating that they have specific functions in these cells [6,7,8,9,10]. For instance, the data indicate that circPVT1 is aberrantly upregulated and might increase the cell growth in OSCC [11]. Besides, circUHRF1 promotes the carcinomatous malevolent performance of OSCC cells [12]. Of note, a recent study suggested that the regulation of hsa_circ_0005615 (circNFATC3) in human cancer cells might alter proliferation and migration *in vitro* [13]. Some reports verified that the dysregulation of circNFATC3 was closely associated with cell malignant behaviors in hepatocellular carcinoma and gastric cancer [14,15]. Meanwhile, the upregulation of circNFATC3 was able to promote cervical cancer tumor development [16]. Nevertheless, the detailed supervisory effect and mechanism of circNFATC3 on OSCC are not clear, which needs further study.

In recent years, increasing research studies have been focused on the regulatory mechanism of circRNA-miRNA-mRNA in tumor progression [17]. The mechanism proposed is that circRNA might function as the competing endogenous RNAs (ceRNAs) to sequester miRNAs away from target mRNAs [18]. Furthermore, miRNAs are a kind of RNA that regulate cellular processes [19,20]. Some miRNAs of the miR-520 family have been testified in human tumors. For example, miR-520d-5p represses gastric cancer growth and miR-520h plays a vital role in breast cancer progression [21,22]. Besides, miR-520h has been verified to inhibit lung cancer cell malignant behaviors [23]. It has been reported that miR-520h was significantly under-expressed in oral tumors [24] but its precise role in OSCC development remains unclear.

Lactate dehydrogenase A (LDHA) is one of the key enzymes involved in glycolysis, which might convert pyruvate to lactate [25]. In the glycolytic pathway, the key role of LDHA is irrevocably changing pyruvate to lactate and converting NADH to NAD+. Of note, LDHA is highly expressed in many types of cancers [26,27]. As a crucial carcinogen, LDHA is particularly closely related to angiogenesis, tumor growth, and epithelial–mesenchymal transition in various tumors [28,29], containing OSCC [30]. In addition, it has been confirmed that overexpression of LDHA might boost OSCC cell proliferation, migration, and drug resistance [31,32].

Using bioinformatics software, it was shown that miR-520h has some binding sites with circNFATC3 or LDHA. Accordingly, we aimed to explore whether circNFATC3 was implicated in OSCC progression via the miR-520h/LDHA axis.

## 2 Materials and methods

### 2.1 Clinical tissue

Forty-six pairs of OSCC and paracancerous tissues were collected from patients who had undergone surgery at Jingmen No.1 People’s Hospital. None of the patients received any treatment before surgery (Table 1).

**Table 1:** Correlation between circNFATC3 expression and clinicopathological parameters of patients with OSCC

Clinical feature		circNFATC3		
	*n*	High (*n* = 23)	Low (*n* = 23)	*P*-Value
Age (years)				0.234
≥60	20	12	8	
<60	26	11	15	
Gender				0.536
Male	30	16	14	
Female	16	7	9	
Tumor size				0.139
<3 cm	21	8	13	
≥3 cm	25	15	10	
TNM stage				0.036^*^
III–IV	27	17	10	
I–II	19	6	13	
Differentiation				0.238
Well/moderate	24	10	14	
Poor	22	13	9	
Lymph node metastasis				0.017^*^
Negative	20	6	14	
Positive	26	17	9	

^*^ *P* < 0.05.


**Ethics approval and consent to participate:** The present study was approved by the ethical review committee of Jingmen No. 1 People’s Hospital. Written informed consent was obtained from all enrolled patients.

### 2.2 Cell lines

Human OSCC cell lines SCC25, HSC3, and SCC15 were used in this study. In addition, the human normal oral keratinocyte cell lines (HOK) were used as a control. SCC25 and SCC15 cells were acquired from American type culture collection (ATCC, Manassas, VA, USA). HSC3 and HOK cells were purchased from Cell Bank, Chinese Academy of Sciences (CAS, Shanghai, China). According to the instructions, SCC25 and SCC15 were cultured in the complete growth medium with 10% fetal bovine serum (FBS; Sigma-Aldrich, St. Louis, MO, USA). All cells were cultured with 5% CO_2_.

### 2.3 qRT-PCR

RNA was extracted with the Trizol reagent (Sigma). Then, the entire RNA of circNFATC3 and LDHA was reverse-transcribed to cDNA using the Prime Script RT reagent kit (Sigma). Meanwhile, miR-520h was reverse transcribed using the miRNA First-Strand Synthesis kit (Sigma). Next, cDNA was used for qRT-PCR with an SYBR Green kit (Sigma). GAPDH and RNU6 (U6) were used as endogenous controls. The primers were as follows: circNFATC3, F: 5′- ACCCTTTACCTGGAGCAAACC-3′ and R: 5′-TGTGGTAAGCAAAGTGGTGT-3′; NFATC3, F: 5′-TCCACCTCCATCTACTTTAACCA-3′ and R: 5′-TTGGGACCACCTAATGGGCT-3′; LDHA, F: 5′-GAGTGGAATGAATGTTGCTGGTGTC-3′ and R: 5′-CCAGGATGTGTAGCCTTTGAGTTTG-3′; miR-520h, F: 5′-TCGCGACAAAGTGCTTCCCT-3′ and R: 5′-GTGCAGGGTCCGAGGT-3′; GAPDH, F: 5′-TCCCATCACCATCTTCCAGG-3′ and R: 5′-GATGACCCTTTTGGCTCCC-3′; U6, F: 5′-CTCGCTTCGGCAGCACATATACT-3′ and R: 5′-ACGCTTCACGAATTTGCGTGTC-3′. Relative expression was processed using the 2^−△△Ct^ method.

### 2.4 RNase R and actinomycin D assay

The SCC25 and HSC3 RNAs and cells were inactivated with RNase R (Sigma) or Act D (Sigma) according to the manufacturer’s instructions. Afterward, the RNA was used to reverse transcribe into cDNA, and the abundances of circNFATC3 and NFATC3 mRNA were examined by using qRT-PCR.

### 2.5 Western blot

Western blot analysis was performed as given by Shang et al. [33]. The antibodies were applied as follows: anti-LDHA (1:1,000; Santa Cruz Biotechnology, Santa Cruz, CA, USA), anti-MMP2 (1:1,000; Cell Signaling Technology), anti-Slug (1:1,000; Cell Signaling Technology), and anti-β-actin (1:5,000; Sigma).

### 2.6 Cell transfection

circNFATC3 expression was stably inhibited by lentiviral with shRNA. Lentivirus sh-circNFATC3 targeting circNFATC3 (sh-circNFATC3-1, sh-circNFATC3-2), and a non-specific control shRNA (sh-NC), miR-520h mimics (miR-520h), anti-miR-520h, controls, oe-LDHA, and vector were constructed by Sangon Biotech (Shanghai, China). These plasmids or oligonucleotides were transferred into SCC25 and HSC3 cells by using Lipofectamine 3000 (Sangon).

### 2.7 Glycolysis metabolism assay

SCC25 and HSC3 cells were planted into 6-well plates. After 24 h cultivation, the concentrations of glucose and lactate were detected by using Glucose Assay Kit and L-Lactate Assay Kit (Sigma), respectively, according to the manufacturer’s instructions.

### 2.8 Cell proliferation assay

SCC25 and HSC3 cells with diverse transfection were planted in 96-well plates. Cell proliferation was measured at 0, 1, 2, and 3 days using an MTT assay (Sigma). The absorbance was detected at 490 nm. Besides, the EdU Apollo Imaging Kit (Sigma) was used following the manufacturer’s instructions.

### 2.9 Flow cytometry assay

SCC25 and HSC3 cells were plated on the 6-well plates. Then, the cell apoptotic was assessed by using an Annexin V-FITC/PI kit (Sigma) following the manufacturer’s instructions.

### 2.10 Wound-healing assay

SCC25 and HSC3 cells were plated on 6-well plates. In simple terms, the cells were scratched with a germ-free pipette tip and then treated with FBS-free media for 24 h. The distance between two cell boundaries were measured.

### 2.11 Transwell assay

Transwell assay was used to investigate the influence of circNFATC3 deletion on cell invasion [34]. The invasive cells were counted under a microscope.

### 2.12 Dual-luciferase reporter assay

The combinative sites between miR-520h with circNFATC3 or LDHA 3′-UTR were analyzed by Starbase. The circNFATC3 or LDHA 3′UTR wild and mutant were manufactured by Sangon (WT-circNFATC3, WT-LDHA 3′UTR or MUT-circNFATC3, MUT-LDHA 3′UTR). The luciferase activity was examined using the Dual-Luciferase Reporter Assay Kit (Promega, Madison, WI, USA).

### 2.13 RIP assay

SCC25 and HSC3 cells were assayed using the Magna RIP kit (Sigma) following the manufacturer’s instructions. Finally, the circNFATC3 and miR-520h were quantified.

### 2.14 Xenograft models

All mice test processes were performed following the guidelines of the Animal Care and Use Committee of the Jingmen No.1 People’s Hospital. SCC25 cells (5 × 10^6^) with sh-circNFATC3 or sh-NC were injected hypodermically into two groups of 4-week-old nude mice (*n* = 6 mice/group, Beijing Vital River Laboratory Animal Technology Co., Ltd., Beijing, China), respectively. The tumor volumes were observed every day and measured every 4 days: volume = (length × width^2^)/2. After 6 weeks, all mice were killed, and tumor nodes were resected for further examination.

### 2.15 IHC assay

The Ki67 (ab92742; 1:1,000; Abcam), Vimentin (ab92547; 1:1,000; Abcam), and c-caspase 3 (ab32351; 1:1,000; Abcam) abundances in the tumor were quantified by using the IHC assay. The explicit examination technique was in accordance with Ma et al. [35]. The sections were observed.

### 2.16 Statistical analysis

All statistics results were from three independent replications and were investigated using SPSS 23.0. Pearson’s correlation analysis was used to quantify the correlation. Shapiro–Wilk test was used to check for normality, and all data conformed to normal distribution. Student’s *t*-test and ANOVA were used to compare the statistical differences. *P* < 0.05 was significant.

## 3 Results

### 3.1 circNFATC3 was markedly augmented in OSCC

**Figure 1:**
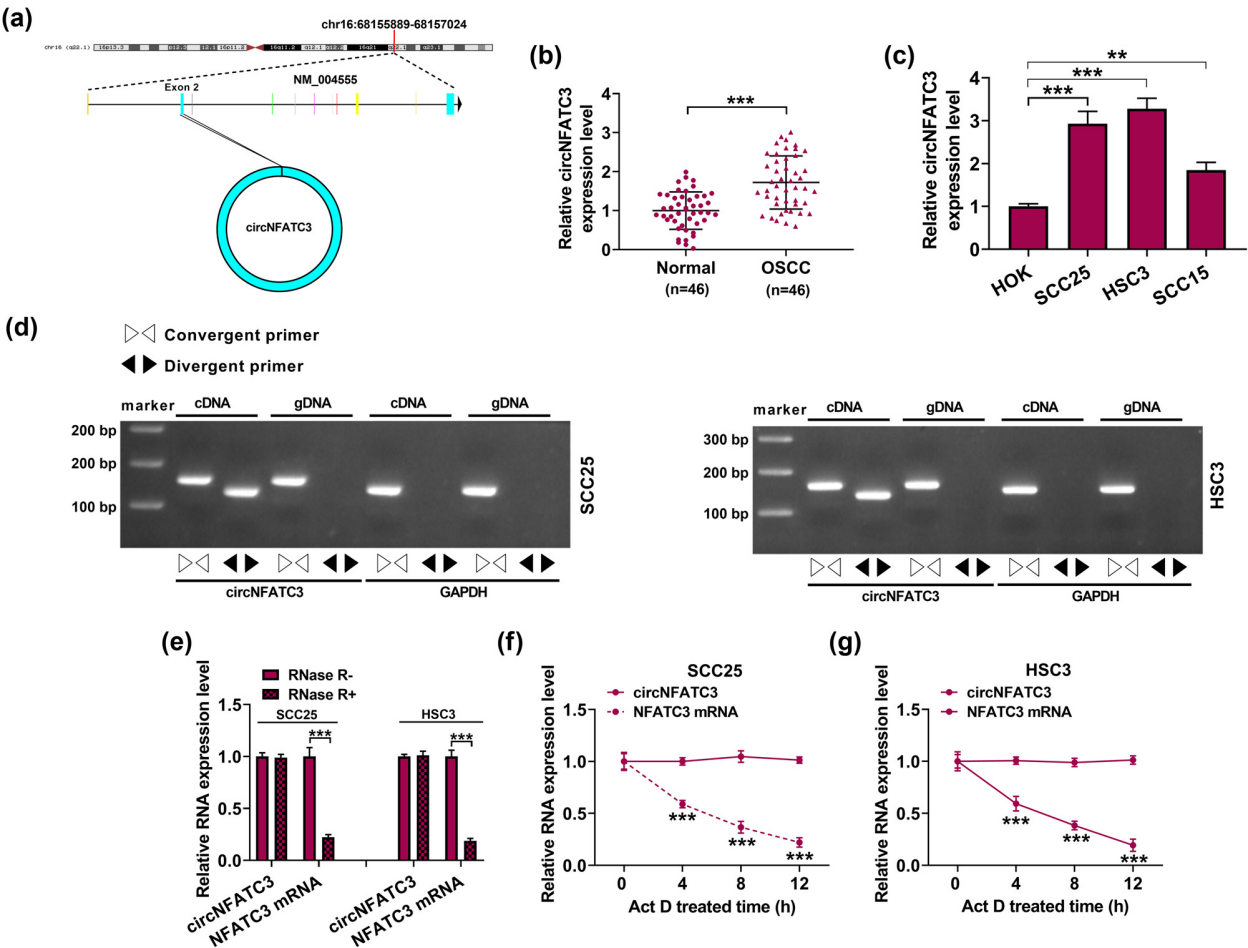
circNFATC3 was enhanced in OSCC. (a) The structure of circNFATC3. (b) The expression of circNFATC3 was detected. (c) The content of circNFATC3 in HOK, SCC25, HSC3, and SCC15 cells was examined. (d) The amplification of circNFATC3 and GAPDH. (e) The relative levels of circNFATC3 and NFATC3 mRNA were determined. (f and g) The circNFATC3 and NFATC3 mRNA levels were measured. **P < 0.01, ***P < 0.001.

**Figure A1:**
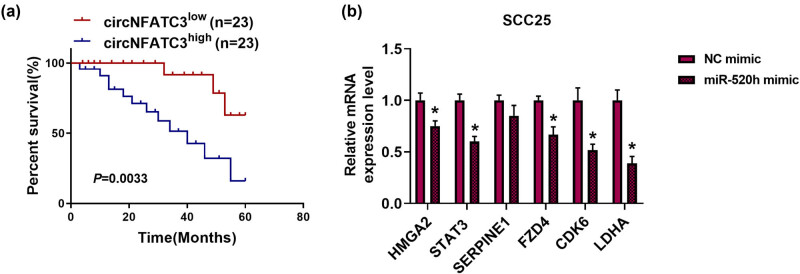
High level of circNFATC3 was correlated with lower overall survival, and the reasons for the introduction of LDHA. (a) The correlation between circNFATC3 expression and overall survival was analyzed. (b) The expression of predicted target mRNAs of miR-520h was detected in SCC25 cells transfected with NC mimic or miR-520h mimic.

Initially, circNFATC3 is located at chr16:68155889-68157024 (Figure 1a). We addressed whether the abundance of circNFATC3 was abnormal in OSCC. circNFATC3 was significantly augmented in OSCC tumor tissues (*n* = 46) and cell lines (SCC25, HSC3, and SCC15) (Figure 1b and c). Based on the median of circNFATC3 expression in OSCC tissues, the patients were divided into high level of circNFATC3 group and low level of circNFATC3 group, and the high level of circNFATC3 was correlated with lower overall survival (Figure A1a). Furthermore, we found that circNFATC3 only enlarged in cDNA through divergent primers but not in gDNA (Figure 1d). Hence, to further explore the structure of circNFATC3, RNase R and Act D enzyme assay was performed to measure the structures of circNFATC3 and NFATC3 mRNA in OSCC cell lines (SCC25 and HSC3). In general, RNase R and Act D do not absorb circRNAs but absorb only linear RNAs [36]. As displayed in Figure 1e–g, NFATC3 mRNA was significantly decreased after digestion by RNase R and Act D when compared with circNFATC3. The results confirmed the cyclic structure of circNFATC3. These outcomes suggested that the circNFATC3 contents were augmented in OSCC and might take effect in OSCC. Besides, the circNFATC3 structure pattern was circRNAs.

### 3.2 Silencing circNFATC3 induced cell apoptosis, while subdued glycolysis metabolism, cell proliferation, migration, and invasion in OSCC cells

**Figure 2:**
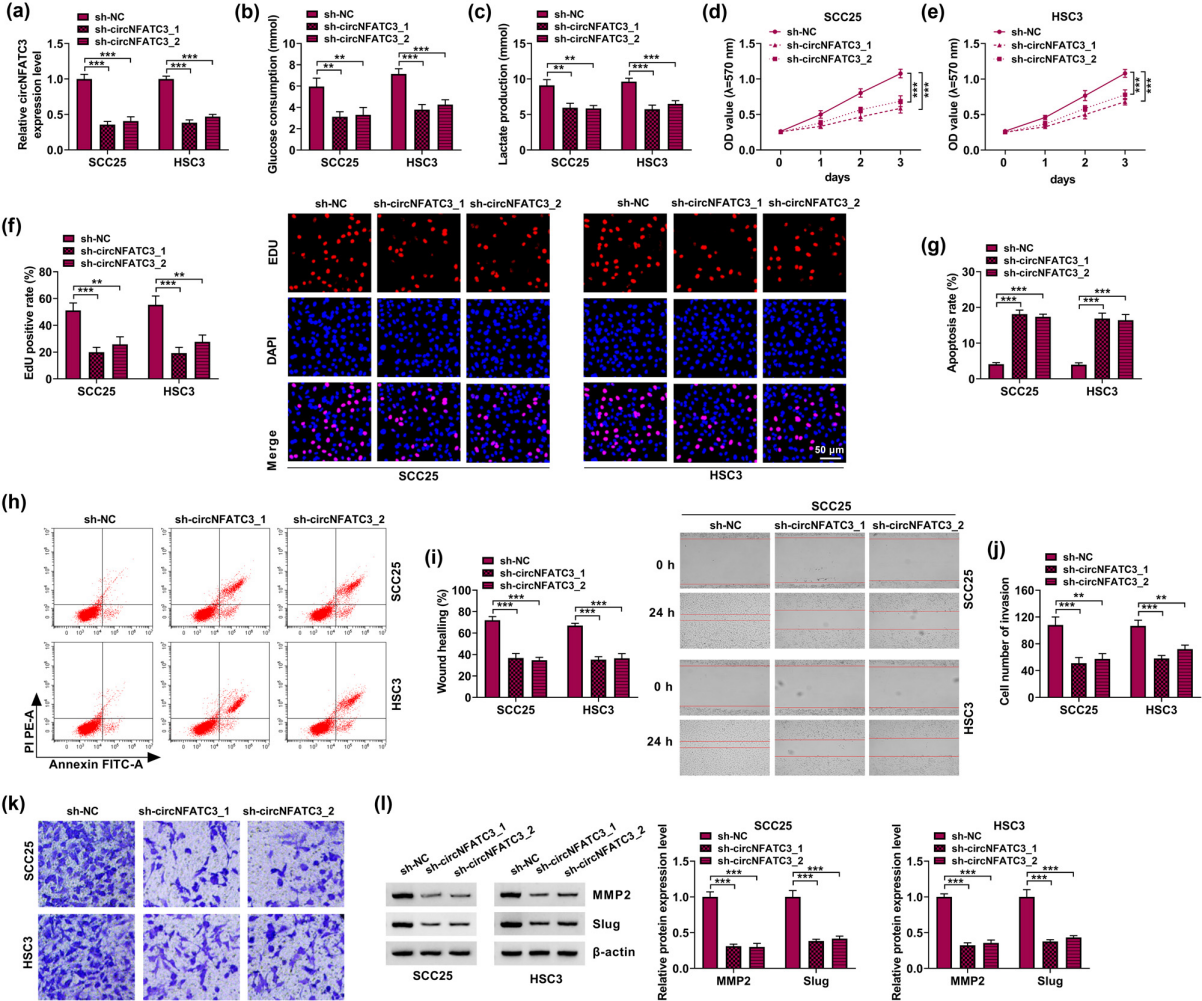
The absence of circNFATC3 lack inhibited OSCC. (a) The silencing efficacy of circNFATC3 was assessed. (b and c) The levels of glycolysis metabolism were examined. (d–f) The impact of circNFATC3 deletion on the proliferation of SCC25 and HSC3 cells was illustrated. (g and h) Flow cytometry assay was carried out to explain the effects of circNFATC3 knockdown on the apoptosis of SCC25 and HSC3 cells. (i) The rate of wound healing was illustrated by the wound healing assay. (j and k) Transwell assay was enforced to investigate the influences of circNFATC3 deletion on cell invasion. (l) The levels of MMP2 and Slug were examined. **P < 0.01, ***P < 0.001.

SCC25 and HSC3 were transfected with sh-circNFATC3 (sh-circNFATC3-1, sh-circNFATC3-2), with sh-NC as the control. The transfection efficiency of sh-circNFATC3 was quantified by qRT-PCR. The results showed that circNFATC3 was significantly decreased in SCC25 and HSC3 cells by sh-circNFATC3 (Figure 2a). In addition, the knockdown of circNFATC3 significantly repressed glycolysis metabolism in SCC25 and HSC3 cells (Figure 2b and c). Next, circNFATC3 reduction notably decreased cell proliferation (Figure 2d–f). Besides, the absence of circNFATC3 encouraged cell apoptosis in SCC25 and HSC3 cells (Figure 2g and h). Subsequently, the absence of circNFATC3 subdued migration and invasion of SCC25 and HSC3 cells (Figure 2i–k). MMP2 and Slug were linked with the migration and invasion of OSCC cells [37,38]. Here, we identified that sh-circNFATC3 transfection conspicuously abridged the MMP2 and Slug levels in SCC25 and HSC3 cells (Figure 2l). Our results indicated that deficiency of circNFATC3 induced cell apoptosis, and suppressed cell glycolysis metabolism, cell proliferation, migration, and invasion of OSCC cells.

### 3.3 MiR-520h targeted circNFATC3 in OSCC cells

**Figure 3:**
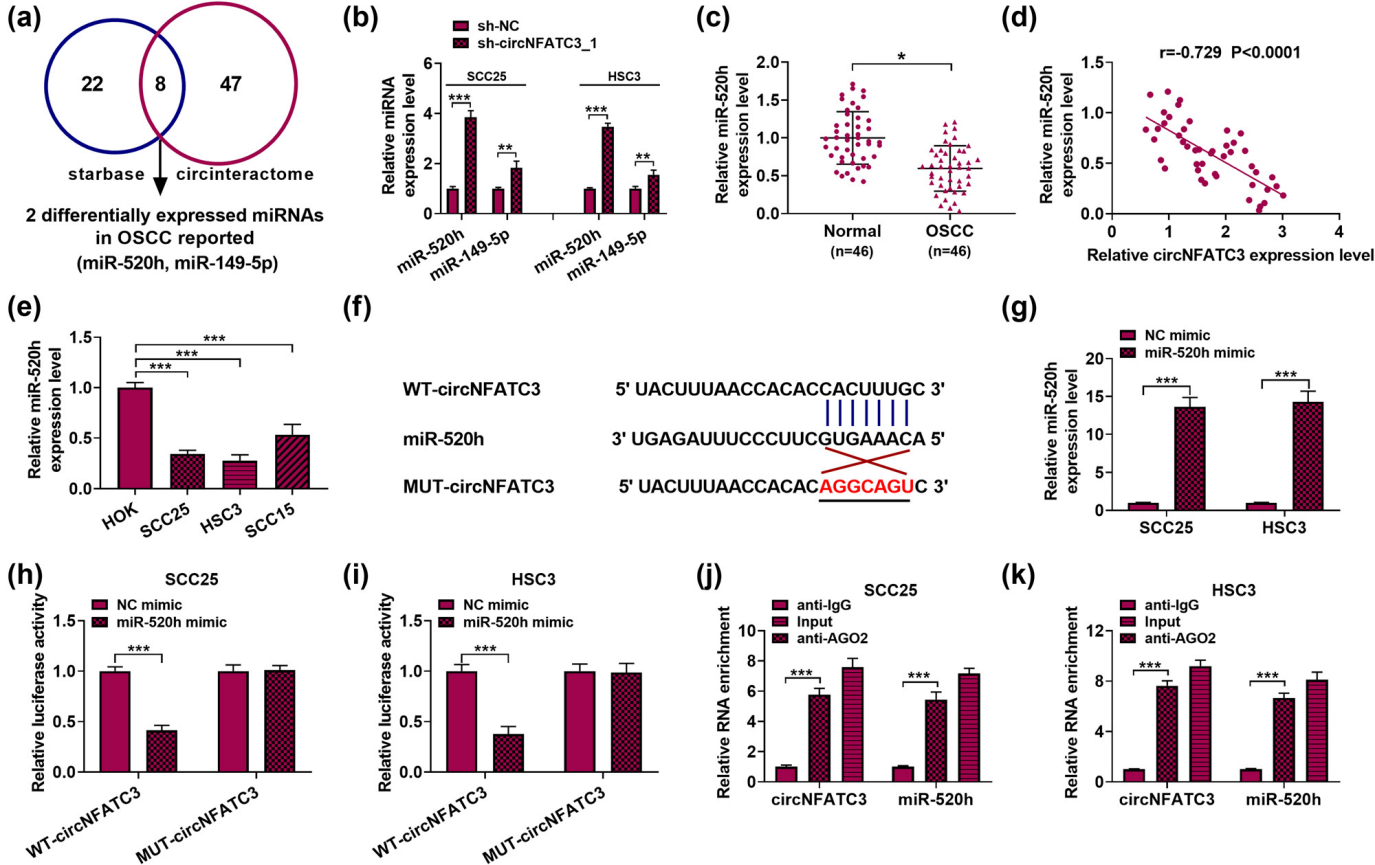
circNFATC3 as a miR-520h sponge. (a) The targeted binding of circNFATC3 and miRNAs was forecast by Starbase and Circinteractome. (b) The effects of knockdown circNFATC3 and miR-520h expression in OSCC cells. (c) The abundance of miR-520h in OSCC tumor tissues (*n* = 46) and paracancerous tissues (*n* = 46) was measured. (d) Pearson’s correlation analysis showed that circNFATC3 was negatively associated with miR-520h in OSCC tumor tissues (*R* = −0.729). (e) The content of miR-520h in OSCC cells was examined. (f) The binding sequence between circNFATC3 and miR-520h was predicted by Targetscan 7.0. (g) The overexpression efficiency of miR-520h in OSCC cells was examined by qRT-PCR. (h and i) Dual-luciferase reporter assay was used to reveal the relationship between circNFATC3 and miR-520h. (j and k) RIP analysis proved that circNFATC3 was abundantly pulled down by anti-Ago2 antibodies when transfected with miR-520h mimics in OSCC cells versus the miR-520h NC and the IgG group. *P < 0.05, ***P < 0.001.

Starbase and Circinteractome were used to forecast the miRNAs of circNFATC3 (among the 8 miRNAs with an intersection, two miRNAs differentially expressed in OSCC were reported: miR-520h and miR-149-5p) (Figure 3a). Then, the relative levels of miR-520h and miR-149-5p in SCC25 and HSC3 after transfection with sh-circNFATC3 and sh-NC were determined by qRT-PCR. Expression analysis showed that the expressions of the two miRNA were notably impaired, and the expression of miR-520h was significantly increased. Therefore, miR-520h was used in the following experiments (Figure 3b). We addressed whether the expression of miR-520h was abnormal in OSCC tissues. The qRT-PCR results indicated that miR-520h was significantly lesser in OSCC tumor tissues (*n* = 46) than that in paracancer tissues (*n* = 46) (Figure 3c). In addition, Pearson’s correlation analysis indicated that miR-520h was negatively correlated with circNFATC3 in OSCC tissues (Figure 3d), and its expression displayed a low expression in OSCC cells relative to their respective controls (Figure 3e). As shown in Figure 3f, there were some combinative sites between circNFATC3 and miR-520h, which were subsequently confirmed by using dual-luciferase reporter assay. Consequences revealed that miR-520h notably increased the abundances of miR-520h in OSCC cells (Figure 3g). In addition, results revealed that the luciferase activity was decreased in OSCC cells co-transfected with WT-circNFATC3 and miR-520h. However, there was no change in luciferase activity after co-transfection with MUT-circNFATC3 and miR-NC (Figure 3h and i). RIP assays further confirmed the targeted relationship of miR-520h and circNFATC3 (Figure 3j and k). The above experimental results showed that there was a clear targeting relationship between miR-520h and circNFATC3. In a word, the results revealed that circNFATC3 served as a miR-520h sponge in OSCC, and it could play a crucial role in OSCC growth.

### 3.4 circNFATC3 contributed to OSCC via miR-520h

**Figure 4:**
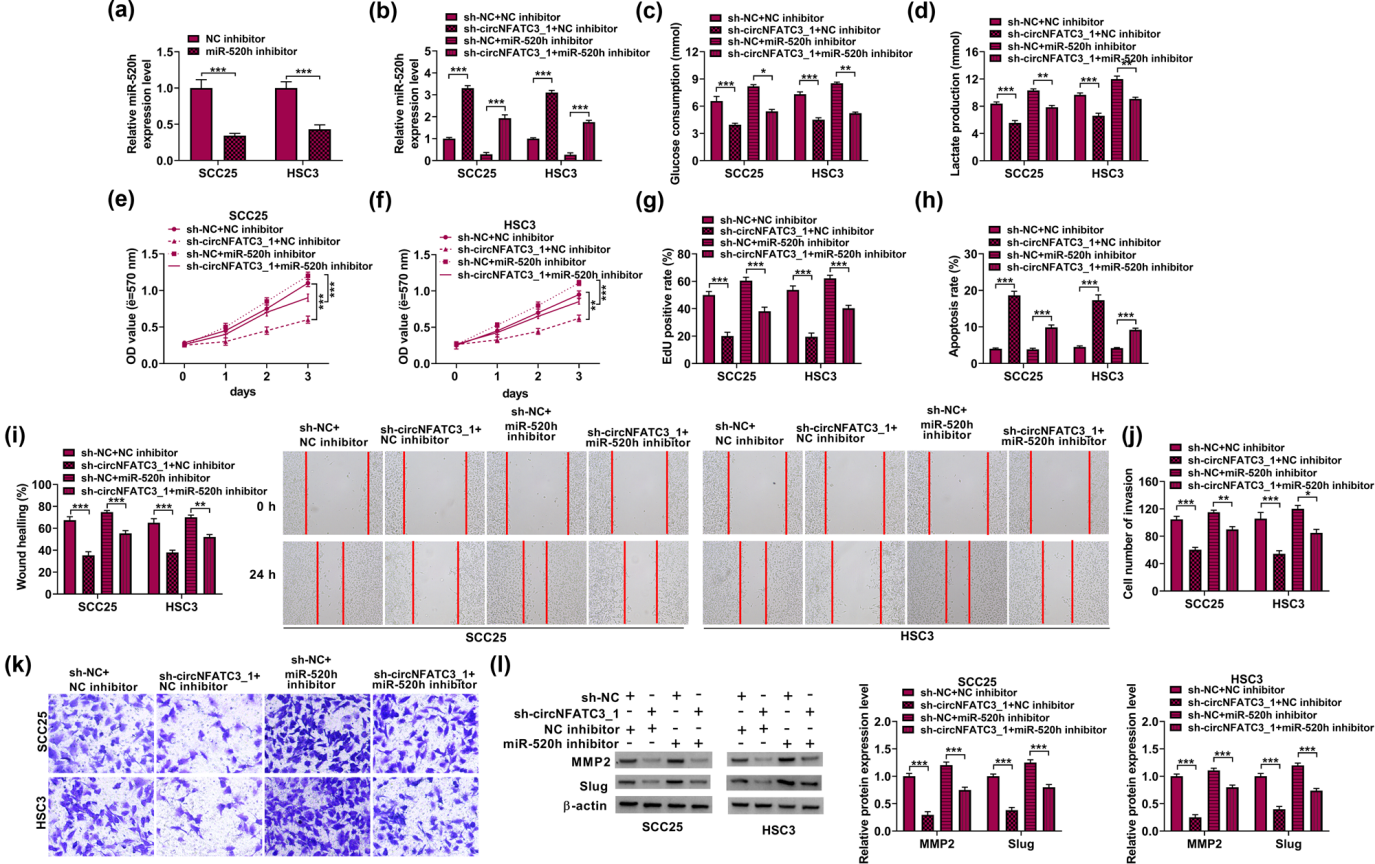
circNFATC3 expedited OSCC via miR-520h. (a) The interfering efficiency of miR-520h was determined in SCC25 and HSC3 cells. (b) The miR-520h expression was examined in SCC25 and HSC3 cells. (c and d) The levels of glycolysis metabolism, (e–g) the level of cell proliferation, (h) the apoptosis, (i) the rate of wound healing, (j and k) the number of invaded cells, and (l) the protein level of MMP2 and Slug were examined. *P < 0.05, **P < 0.01, ***P < 0.001.

To further study the effects of circNFATC3 and miR-520h on OSCC progression, we first identified the interfering efficiency of miR-520h inhibitors. Primarily, qRT-PCR indicated that the abundances of miR-520h were diminished by miR-520h inhibitors in SCC25 and HSC3 cells (Figure 4a). Meanwhile, we found that circNFATC3 knockdown increased the expression of miR-520h in SCC25 and HSC3 cells, while this influence was diminished by miR-520h inhibitor (Figure 4b). Afterward, circNFATC3 silencing significantly repressed glycolysis metabolism in SCC25 and HSC3 cells, but this impact was partially attenuated by miR-520h knockdown (Figure 4c and d). Subsequently, miR-520h knockdown decreased the inhibition effects of circNFATC3 silencing on cell proliferation in SCC25 and HSC3 cells (Figure 4e–g). On the other hand, the miR-520h inhibitor sectionally restored the promotion effect of circNFATC3 inhibition on cell apoptosis (Figure 4h). Consistent with these observations, miR-520h inhibitors restrained the suppression impacts of circNFATC3 silencing on cell migration and invasion in SCC25 and HSC3 cells (Figure 4i–k). We further performed Western blot assay, which showed that miR-520h inhibitors restrained the repression impacts of circNFATC3 silencing on the expression of MMP2 and Slug in SCC25 and HSC3 cells (Figure 4l).

### 3.5 MiR-520h targeted LDHA in SCC25 and HSC3 cells

**Figure 5:**
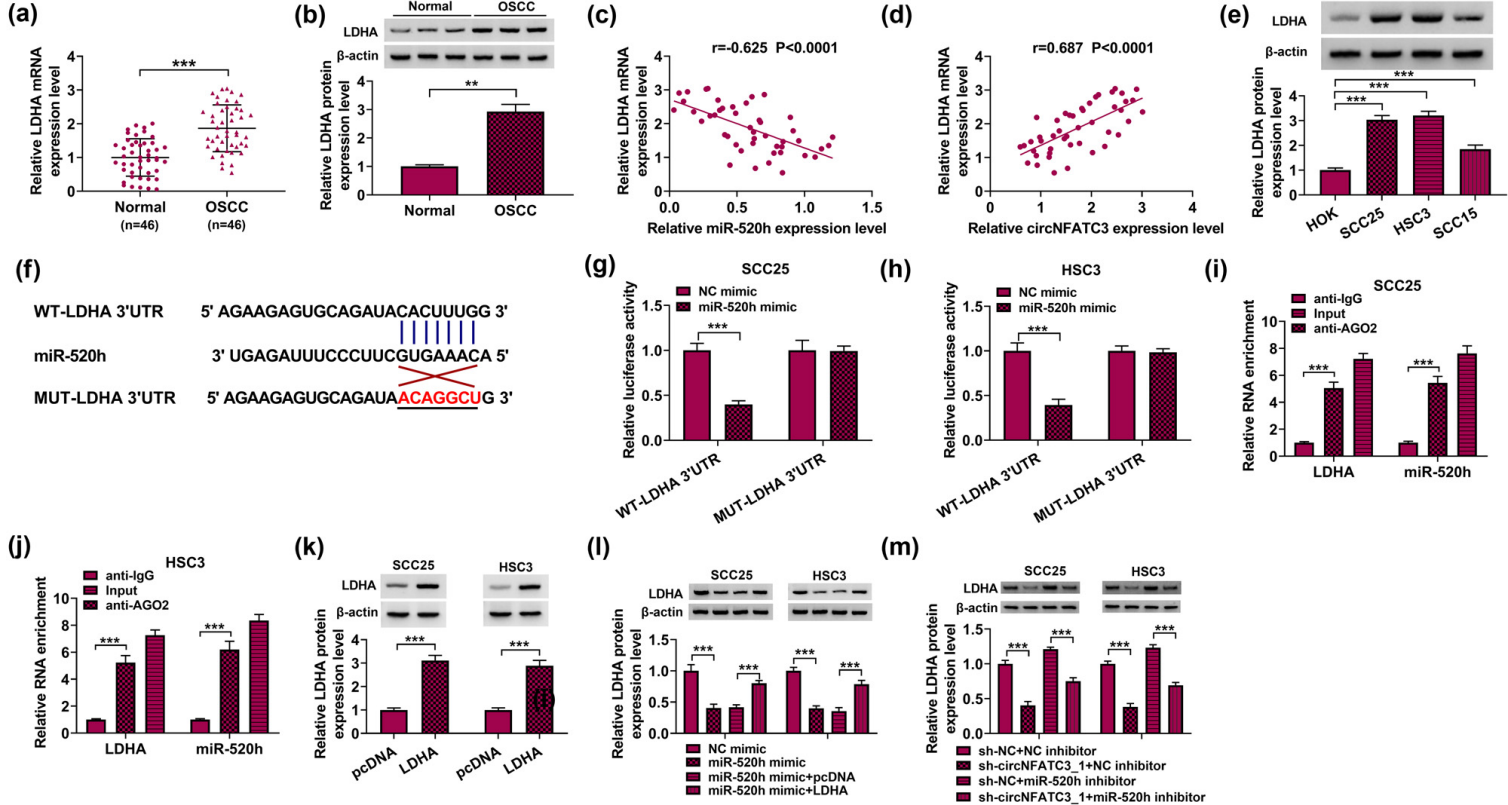
miR-520h targets LDHA in SCC25 and HSC3 cells. (a) The contents of LDHA in OSCC tumor tissues (*n* = 46) and paracancerous tissues (*n* = 46) were quantified. (b) The abundances of LDHA in OSCC tumor tissues and paracancerous tissues were measured. (c) Pearson’s correlation analysis showed that miR-520h was negatively linked with LDHA in OSCC tumor tissues (*R* = −0.625). (d) Pearson’s correlation analysis showed that circNFATC3 was positively linked with LDHA in OSCC tumor tissues (*R* = 0.687). (e) The level of LDHA in OSCC cells was measured. (f) The connective site between miR-520h and LDHA. (g and h) The association of miR-520h and LDHA. (i and j) RIP analysis proved the relationship between miR-520h and LDHA. (k) The effectiveness of LDHA overexpression was ensured by Western blot analysis. (l and m) The content of LDHA was distinguished. **P < 0.01, ***P < 0.001.

First, the expression of some predicted target mRNAs of miR-520h was detected, and LDHA was the most significantly downregulated by miR-520h (Figure A1b). Besides, LDHA levels were significantly increased in OSCC tissues compared to paracancer tissues (Figure 5a and b). Besides, Pearson’s correlation analysis showed that miR-520h was negatively correlated with the LDHA, but circNFATC3 was positively correlated with the LDHA (Figure 5c and d). Furthermore, LDHA abundances were significantly enhanced in OSCC cells (Figure 5e). The target gene of miR-520h was confirmed in this part. StarBase v2.0 online database was used to envisage the combinative sites of miR-520h in LDHA 3′-UTR (Figure 5f). Dual-luciferase reporter assay indicated that the luciferase activity in the WT-LDHA 3′-UTR group was significantly reduced by miR-520h. Yet the luciferase activity of the MUT-LDHA 3′-UTR group was not dramatically altered by miR-520h (Figure 5g and h). RIP assay results confirmed the interaction between miR-520h and LDHA (Figure 5i and j). The above experimental results showed that there was a clear targeting relationship between miR-520h and LDHA. Western blot assay was used to reveal that the protein expression of LDHA was memorably upregulated due to LDHA overexpression (Figure 5k). We further performed a Western blot assay, which showed that LDHA reversed the inhibition influences of miR-520h mimics or reduced the abundances of LDHA in SCC25 and HSC3 cells (Figure 5l). At the same time, we found that miR-520h inhibitor attenuated the suppression impacts of sh-circNFATC3 or abridged the contents of LDHA in SCC25 and HSC3 cells (Figure 5m).

### 3.6 MiR-520h curbed the OSCC via LDHA

**Figure 6:**
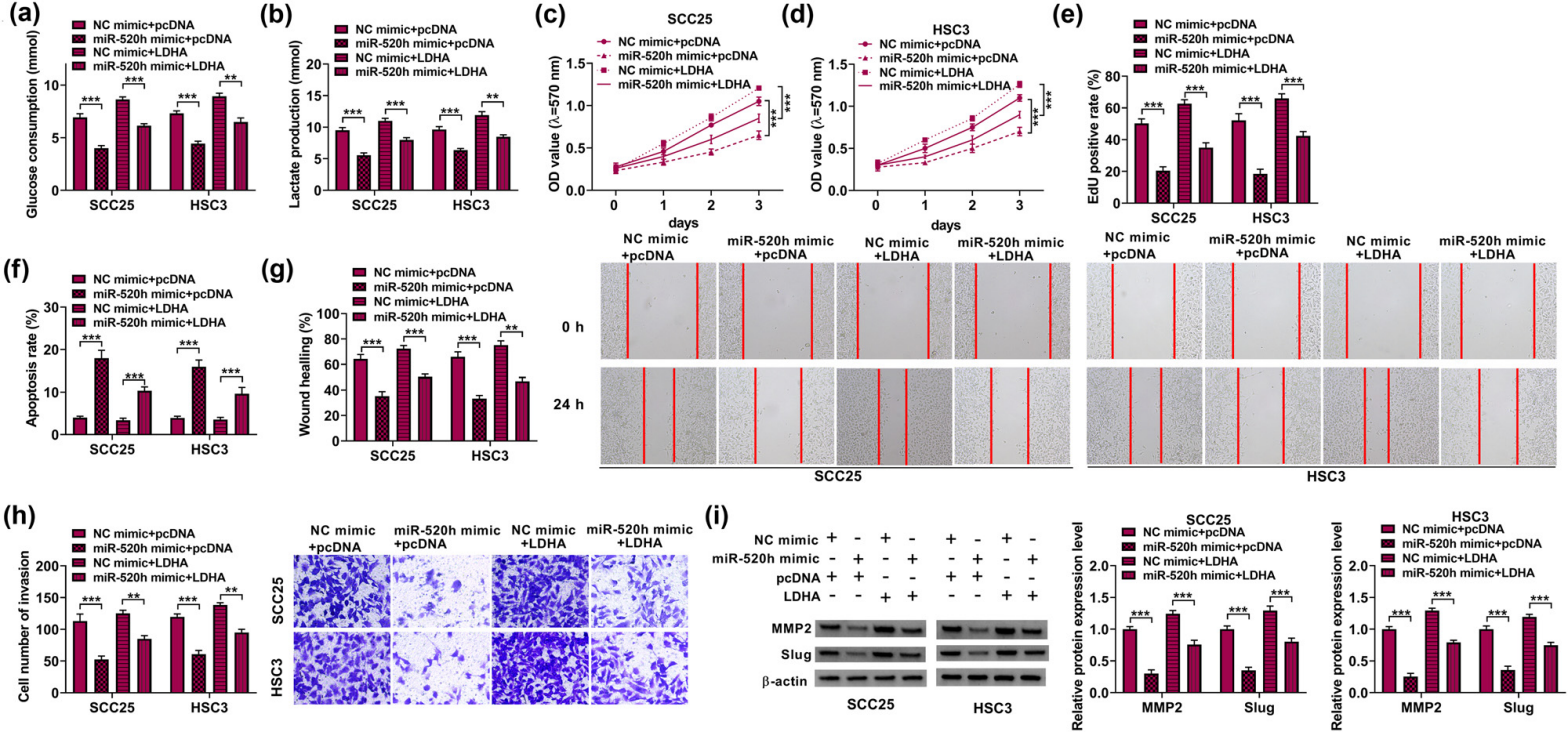
miR-520h adjusted OSCC via LDHA. (a and b) The levels of glycolysis metabolism, (c–e) cell proliferation, (f) apoptosis, (g) the rate of wound healing, (h) the invaded cells, and (i) the protein level of MMP2 and Slug were examined. **P < 0.01, ***P < 0.001.

To further explore whether miR-520h inhibited the progression of OSCC cells by targeting LDHA, we transfected the miR-520h mimic, NC mimic, miR-520h mimic + pcDNA, and miR-520h mimic + LDHA in SCC25 and HSC3 cells. Afterward, the miR-520h mimic significantly repressed glycolysis metabolism in SCC25 and HSC3 cells, whereas this influence was partially decreased by LDHA (Figure 6a and b). MiR-520h reduced the proliferation of SCC25 and HSC3 cells, but there was a positive conclusion of LDHA (Figure 6c–e). Moreover, by flow cytometry analysis, we established that miR-520h mimics expedited cell apoptosis in SCC25 and HSC3 cells, and this influence was inhibited by LDHA (Figure 6f). In addition, the migration and invasion of SCC25 and HSC3 cells were suppressed by the miR-520h mimic; however, LDHA overexpression could partially abolish these impacts. (Figure 6g and h). We further performed the Western blot assay, which showed that the expression of MMP2 and Slug in SCC25 and HSC3 cells were diminished by miR-520h mimic, whereas LDHA overexpression could partly decrease these influences (Figure 6i). In brief, all these results demonstrated that miR-520h adjusted the cell glycolysis metabolism, viability, colony formation, cell migration, and apoptosis of OSCC cells via LDHA.

### 3.7 circNFATC3 contributed to OSCC by regulating LDHA

**Figure 7:**
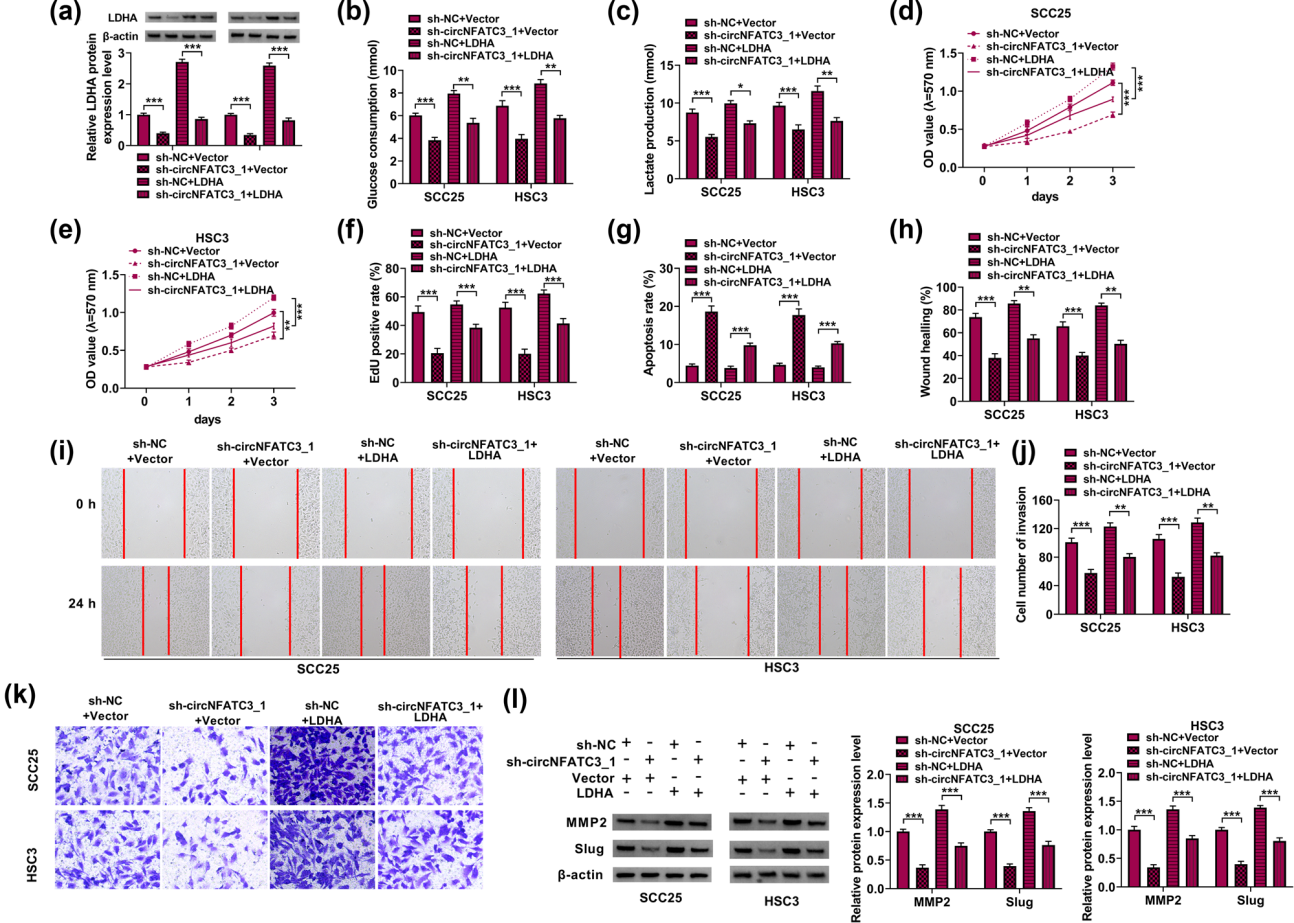
circNFATC3 contributed to OSCC by regulating LDHA. (a) The LDHA expression was examined in SCC25 and HSC3 cells. (b and c) The levels of glycolysis metabolism, (d–f) the level of cell proliferation, (g) apoptosis, (h and i) the rate of wound healing, (j and k) the number of invaded cells, and (l) the protein levels of MMP2 and Slug were examined. *P < 0.05, **P < 0.01, ***P < 0.001.

Primarily, qRT-PCR indicated that the LDHA level was diminished by the absence of circNFATC3 but increased by LDHA (Figure 7a). Afterward, circNFATC3 silencing significantly repressed glycolysis metabolism in SCC25 and HSC3 cells, but this impact was partially attenuated by enhanced LDHA (Figure 7b and c). Subsequently, the enhanced LDHA decreased the inhibition effects of circNFATC3 silencing on cell proliferation (Figure 7d–f). On the other hand, LDHA sectionally restored the promotion effect of circNFATC3 inhibition on cell apoptosis (Figure 7g). Consistent with these observations, LDHA restrained the suppression impacts of circNFATC3 silencing on cell migration and invasion in SCC25 and HSC3 cells (Figure 7h–k). We further performed the Western blot assay, which showed that LDHA restrained the inhibition impacts of circNFATC3 silencing on the expression of MMP2 and Slug in SCC25 and HSC3 cells (Figure 7l).

### 3.8 The absence of circNFATC3 harmed tumor growth *in vivo*

**Figure 8:**
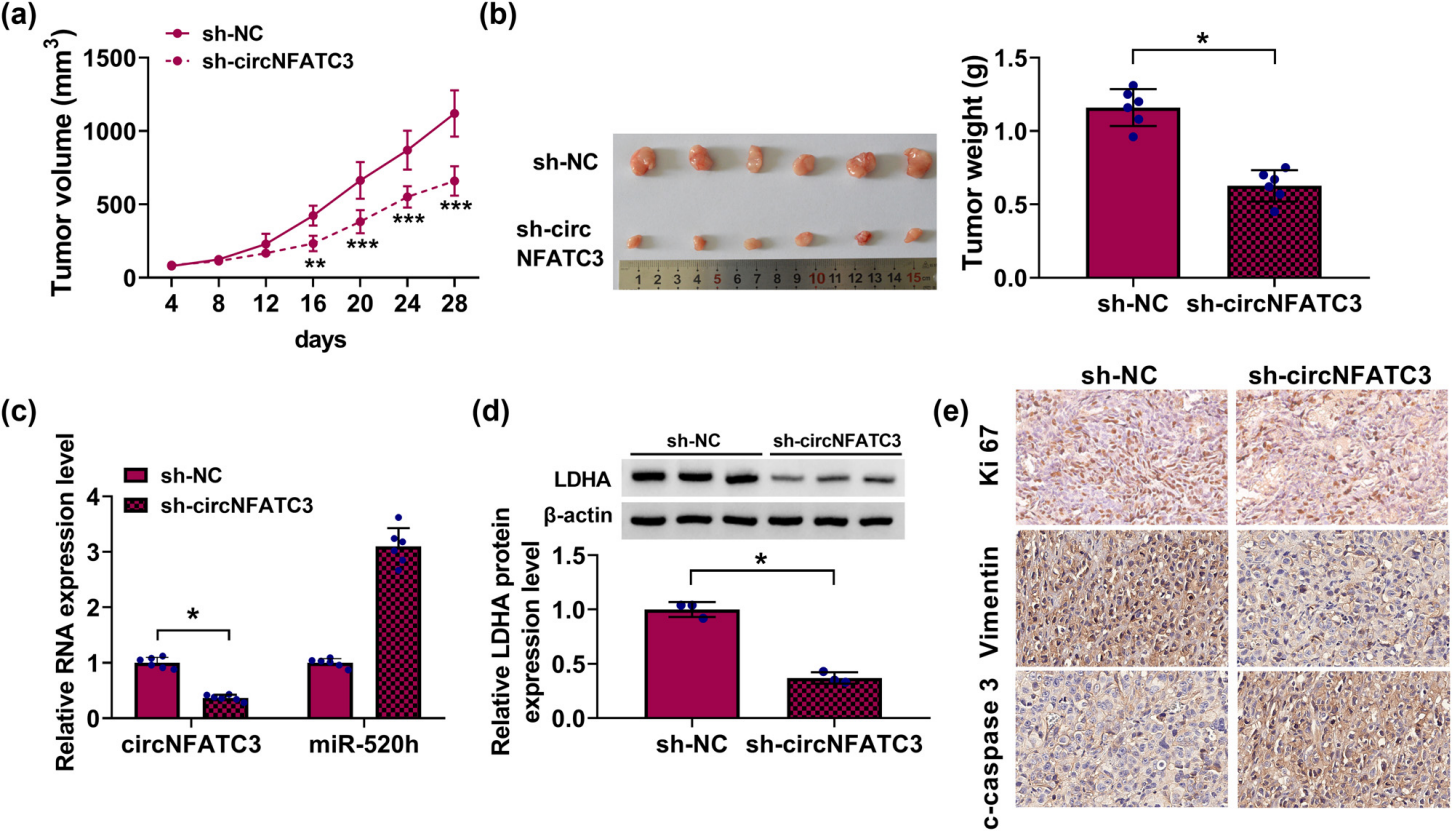
circNFATC3 knockdown restricted tumor growth. (a and b) Tumor volumes and weight was measured. (c) The contents of circNFATC3 and miR-520h were measured. (d) The LDHA level was quantified. (e) IHC analysis was applied to measure Ki67, Vimentin, and c-caspase 3 contents. *P < 0.05.

As presented in Figure 8a and b, the SCC25 cells with sh-circNFATC3 or negative control (sh-NC) were hypodermically injected into athymic nude mice, respectively. Subsequently, we established that intratumoral injection of sh-circNFATC3 blocked tumor volume and weight. Additionally, circNFATC3 and LDHA contents were abridged, although miR-520h was amplified in the sh-circNFATC3 group (Figure 8c and d). The outcomes of IHC revealed that the Ki67 and Vimentin abundances were lesser, but c-caspase 3 contents were higher in the sh-circNFATC3 group, which indicated that the absence of circNFATC3 subdued tumor growth and invasiveness but promoted apoptosis *in vivo* (Figure 8e). These results indicate the absence of circNFATC3 subdued xenograft tumor growth via the miR-520h/LDHA axis.

## 4 Discussion

Herein, we aimed to clarify the function and mechanism of circNFATC3 in OSCC malignancy. In the current research, circNFATC3 might act as an oncogene in OSCC via the miR-520h/LDHA axis.

OSCC is a type of cancer that accounts for about 90% of all oral malignancies and comprises cancers in the mouth and oropharynx [39]. Worldwide, the incidence ranks OSCC among the top three of all cancers in some Asia-Pacific countries [40]. Although the OSCC is frequently identified at an advanced stage, the 5-year overall survival rate of OSCC cases is appraised to be 50–60% [41]. Consequently, it is of great significance to study the molecular mechanism of the pathogenesis of OSCC in this article. This research clarified that circNFATC3 facilitated OSCC progression via the miR-520h/LDHA axis. By competitively binding to miR-520h, circNFATC3 could relieve the latter’s transcriptional inhibition of LDHA protein and improve the transcription and protein expression of LDHA, thus promoting the development of OSCC cells.

Previous studies discovered that some circRNAs served as an imperative part in the advancement of OSCC. Liu et al. uncovered that circIGHG promoted OSCC by tempting epithelial-to-mesenchymal changeover [42]. Su et al. reported that circPHIP supported OSCC by adjusting PHIP and ACTN4 expression, which was related to tumor metastasis and the TNM stage [43]. Additionally, circBICD2 knockdown suppressed OSCC cell proliferation, glycolysis, migration, and invasion but facilitated apoptosis [44]. In this study, we found that circNFATC3 acted as a tumor promoter and its deficiency might induce OSCC cell apoptosis and subdue cell glycolysis metabolism, cell proliferation, migration, and invasion. In addition, our study on mice further discovered that knockdown circNFATC3 attenuated tumor growth. CircRNAs might impact protein-coding genes, which are competitive sponging for miRNAs. For example, circIGHG, circBICD2, and circPHIP could promote OSCC by sponging miR-142-5p [42,43,44]. In this study, circNFATC3 was exposed to elevate OSCC via miR-520h.

MiR-520h facilitated protein kinase 2 (DAPK2), which was associated with cell death [22]. Moreover, miR-520h affected the progression of lung cancer [23]. Furthermore, miR-520h also adjusted metastasis in cervical cancer [45]. These conclusions uncovered that miR-520h played a key role in the progression of human cancers. In this experiment, we proved that miR-520h regulated the advancement of OSCC. We also revealed the suppressive character of miR-520h in glycolysis metabolism, proliferation, and cell migration by targeting LDHA. The results indicated that miR-520h might link with the progress of OSCC, in agreement with C. M. Su et al.’s findings.

It was previously reported that LDHA promoted tumor growth and enhanced cancer cell metabolism [46]. LDHA silencing exposed a connection between glycolysis and tumor preservation [47]. Knockdown of LDHA also restrained metastasis of hepatocellular carcinoma cells and tumor growth [48]. In this study, LDHA contents were significantly increased in OSCC compared to that in paracancerous tissues and cells. In addition, we found that miR-520h repressed cell glycolysis metabolism, proliferation, cell migration, and invasion, and these influences were weakened by LDHA overexpression. We revealed that the absence of miR-520h alleviated the inhibitory conclusion of the absence of circNFATC3 on LDHA contents in OSCC cells. These results further supported the regulatory role of circNFATC3/miR-520h/LDHA in OSCC cells. This study had some insightful findings though it still had some limitations. For instance, the results and conclusions obtained from commercial cell lines are not fully representative of the actual conditions, which also lacks clinical statistical support. In the next step, we will further verify the role of circNFATC3 in clinical application.

In summary, the results indicated that circNFATC3 and LDHA levels increased while miR-520h content decreased in OSCC. Besides, our research first demonstrated that the absence of circNFATC3 suppressed OSCC cell glycolysis metabolism, proliferation, cell migration, and invasion by regulating the miR-520h/LDHA axis. This mode should be further confirmed by clinical research in the future. We trust that this information might deliver a new contrivance for improving OSCC treatments.
